# Association of the Trough, Peak/Trough Ratio of Imatinib, Pyridine–N-Oxide Imatinib and ABCG2 SNPs 34 G>A and SLCO1B3 334 T>G With Imatinib Response in Egyptian Chronic Myeloid Leukemia Patients

**DOI:** 10.3389/fonc.2020.01348

**Published:** 2020-08-19

**Authors:** Mervat M. Omran, Raafat Abdelfattah, Heba S. Moussa, Nelly Alieldin, Samia A. Shouman

**Affiliations:** ^1^Pharmacology Unit, Cancer Biology Department, National Cancer Institute, Cairo University, Cairo, Egypt; ^2^Medical Oncology Department, National Cancer Institute, Cairo University, Cairo, Egypt; ^3^Clinical Pathology Department, National Cancer Institute, Cairo University, Cairo, Egypt; ^4^Medical Statistics Department, National Cancer Institute, Cairo University, Cairo, Egypt

**Keywords:** imatinib, pyridine-N-oxide imatinib, P/T ratio, response, SNP, ABCG2, CML

## Abstract

Imatinib mesylate (IM) is highly efficacious in the treatment of chronic myeloid leukemia (CML). Therapeutic drug monitoring and pharmacogenetic screening are affirmed for better management of IM therapy. The goal of this study was to gain a greater mechanistic understanding of the factors controlling variability in IM level and its relation to the response. One hundred and two patients with CML at chronic phase were recruited in this study. Blood samples were withdrawn at least 30 days after drug administration, and trough and peak concentrations of imatinib, N-des-methyl imatinib, and pyridine-N-oxide imatinib were determined by HPLC/MS/MS. Genetic polymorphism of the genes ABCG2 SNPs 34 G>A and 421C >A; ABCB1 SNPs 2677 G>A/T, 1236 C>T, 3435 C>T; SLCO1B3 SNPs 334 T>G and CYP3A5 were studied using PCR-RFLP technique. Our study presented significant higher trough IM (1,281 ± 578 ng/ml), lower Peak/Trough ratio, clearance (Cl), and elimination rate constant, k_e_, among patients who achieved favorable responses (*N* = 64) than those for patients who suffered unfavorable response (*N* = 37). The P/T ratio was the only significant independent factor affecting response, as the P/T ratio increased by one, the risk of unfavorable response increased by more than double as compared to favorable response with 95% CI (1.28–3.92, *P* = 00.005). Moreover, like the results of IM, the trough concentration of Pyridine-N-oxide imatinib was significantly higher (*P* = 0.01) and its P/T ratio was significantly lower (*P* = 0.008) in patients achieved favorable response than those without. The wild GG genotype of the ABCG2.34 G>A gene was associated with favorable response (*P* = 0.01), lower Cl, K_e_ and high plasma IM trough level than both (AA+GA) genotypes. ABCG2.421C >A (CC) genotype had a significantly higher plasma peak of IM, N-des-methyl imatinib and higher C_ss_. The GG and TG alleles of the SLCO1B3.334 T>G gene were significantly correlated to favorable response, while the wild allele TT was linked to unfavorable response (*P* = 0.03). In conclusion, the trough and P/ T ratio for both IM and Pyridine-N-oxide imatinib, in addition to Polymorphism of ABCG2 SNPs 34 G>A and SLCO1B3.334 T>G gene, is a good predictor for response of IM in CML Egyptian patients.

## Introduction

Chronic myeloid leukemia (CML) is characterized by a chromosomal abnormality, the Philadelphia [Ph] chromosome, which resulted in a unique molecular event, *BCR-ABL1* oncogene, which encodes the chimeric BCR-ABL1 protein with constitutive kinase activity (1, 2). In the absence of treatment, CML is an unavoidable and fatal disease. Imatinib mesylate (IM) is a tyrosine kinase inhibitor that selectively inhibits the BCR-ABL1 oncoprotein and induces effective and safe durable cytogenetic responses in most patients (3). Imatinib has been found effective in the chronic and accelerated phases of CML, as well as in blast crisis (4). The estimated rate of complete cytogenetic response (CCyR) to IM at 18 months was 76%. The 5-year follow-up analysis specified an estimated 87% cumulative CCyR and an estimated overall survival of 89% among 553 patients who received IM as first-line therapy (5).

Imatinib pharmacokinetics (PK) is characterized by rapid and complete oral bioavailability (98%) and a proportional dose-exposure relationship. It is used once daily as its terminal half-life is ~20 h (6). Despite the significant efficacy and better clinical responses and the favorable pharmacokinetic properties of IM, there are cases of suboptimal responses, developing drug resistance, or undergoing relapse after initial success. Therefore, understanding the key contributors to interpatient variability in imatinib disposition is essential for better treatment outcome. Data indicate the important roles of pharmacokinetic, pharmacogenetic in IM efficacy, as well as the initial therapeutic response, and for the time to progression (7). Imatinib is mostly metabolized by hepatic cytochrome P450 enzymes system, mainly CYP3A4 and CYP3A5, which exhibit widely variable activity among different individuals leading to high interpatient variability during IM exposure in CML patients (8). N-des-methyl imatinib is an active metabolite of IM, which is pharmacologically active but 3–4 times less cytotoxic than IM, and its plasma level in patients represents ~20% of the parent drug (9). Two N-oxide metabolites of IM, imatinib pyridine-N-oxide, and imatinib piperdine-N-oxide, have also been identified in patient urine 2 h post-dose but were not observed at 24 h after dosing (10).

One of the patient factors that are probable to be relevant for the observed differences in IM pharmacokinetics is the contribution of single nucleotide polymorphisms in genes related to IM absorption, distribution, metabolism, and excretion. Imatinib uptake is mediated by the human organic anionic transporter OATP1B3 (SLCO1B3 gene product) (11). It is a substrate of adenosine triphosphate binding cassette ATP as efflux transporters such as ABCB1 and ABCG2 (12). Imatinib is mostly metabolized by the cytochrome P450 (CYP) proteins CYP3A4 and CYP3A5 (13). This study aimed at investigating the impact of pharmacokinetic and pharmacogenetic in the clinical response of IM in Egyptian patients with CML.

## Patients and Methods

### Design and Sampling

This study was designed as an observational study. A total of 102 patients from the Hematological Outpatient Clinic of the National Cancer Institute, Cairo University, were recruited in this study. The eligible patients were diagnosed with CML, age ≥ 18 years, on IM treatment for at least 30 days (to reach the steady state), and not more than 38 days, with mean and standard deviation of the timepoint of sample collection at 32 ± 2.44 and median (range) 31 (14–22) days. The excluded patients include those with known sensitivity to IM and patients with a severe medical condition that prohibited participation in the study. Plasma level of peak, trough, and peak/trough (P/T) ratio of IM, and its main metabolites, N-des-methyl imatinib, and Pyridine-N-oxide imatinib were determined. Single nucleotide polymorphisms (SNP) of genes involved in IM uptake and efflux transporters and metabolism genes were assessed using restriction fragment length polymorphism (RFLP). In addition, different types of toxicities were monitored. The time of the study was continued for 2 years from the start of sampling time. The study protocol was approved by the Institution Review Board (IRB) of the National Cancer Institute of Cairo University, Egypt, and written informed consent was obtained according to the Declaration of Helsinki from all patients with acceptance number IRB00004025.

### Response Assessment

Pretreatment, cytogenetic analyses of bone marrow or peripheral blood for Philadelphia [Ph] chromosome was done. A minimum of 20 metaphases was required to be examined for a patient to be classified as Ph-positive. The response of the patient to the treatment can be tracked by cytogenetic (karyotype and/or Fish) and molecular (by detecting the presence of BCR-ABL1 mRNA metaphases) that identify the optimal and suboptimal response, and treatment failure, based on molecular and cytogenetic response over the course of treatment (23). According to ELN guidelines, the clinical effect was evaluated at 12 months after IM therapy. Patients with favorable responses include those who achieved either complete cytogenetic response (CCyR) or major molecular response (MMR). The CCyR is defined as 0% Ph metaphase cells of 20 examined field and MMR, subjects who achieved CCyR as 3 or more log reduction in BCR-ABL/BCR ratio by polymerase chain reaction (PCR) assay relative to a standardized baseline derived from the median ratio for 30 untreated patients with chronic-phase CML (24).

### Chemicals

Imatinib mesylate (Enzo, NY, USA), N-des methyl imatinib, Pyridine-N-oxide imatinib, palonosetron hydrochloride were purchased from Santa Cruz Biotechnology (TX, USA), methanol for HPLC 99.9%, 2-propanol (Riedel-deHaën, Honeywell, Germany), formic acid for mass spectrometry, 98%, ethanol (Sigma-Aldrich, Steinheim, Germany), Agarose A (Biobasic, Ontario, Canada), Tris-acetate -EDTA(TAE) (Invitrogen Life Technologies, NY, USA). In addition, DNA ladder (Solis BioDyne, Estonia), PCR Master Mix kit (2X) (Thermo Scientific, IL, USA), Restriction enzymes: RsaI, Acl I, Dde I, Mbo I, AluI (Promega, Madison, MI, USA), and Bse3D I, Bsu R I, Bst 4C I, Bse 3D I (SibEnzyme Ltd, Russia) were obtained.

### Pharmacokinetic Analysis

#### Samples Preparation

After approval of IRB and the individual consent, whole blood samples were collected into EDTA-containing tubes just before drug administration (trough sample), and 2 h after IM administration (peak sample). The plasma was separated by centrifugation at 2,500 × g for 10 min, 400 ul of plasma was transferred to a glass tube and was spiked with 40 μL of the internal standard (IS) stock solution (2 ug/ml), then 1,200 ul of methanol was added. The mixture was mixed by vortex and then centrifuged at 10,000 × g at 4°C for 10 min. The clear supernatant was transferred to HPLC autosampler vials and 10 μl was injected onto the LC/MS/MS system following the method of Titier et al. (25).

#### LC-MS-MS Instrumentation and Operating Conditions

The LC-MS-MS system consisted of a ABSCIEX Q TRAP 3200 mass spectrometer (ABSCIEX, Germany) equipped with an electrospray ionization (ESI) interface coupled to an Agilent 1200 HPLC system (Agilent Technologies, CA, USA) with a quaternary gradient pump (Agilent 1,260 infinity) and an autosampler (Agilent 1,260 infinity). Data acquisition was performed with analyst 4.0 software (ABSCIEX). The separation was performed using Agilent pro shell EC, C18 (5 μm, 50 × 4.6 mm) reversed-phase analytical column (Agilent, CA, USA). The mobile phase consisted of 0.1% formic acid in methanol/water (55:45, v/v) and pumped at a flow rate of 700 μl/min. The overall run time was 6 min. The mass spectrometer was operated in the positive ESI mode at a temperature of 350°C. The calculation is done by the Multiquant software program. Quantification was performed with multiple reaction monitoring (MRM) and the following ion transitions: m/z 494:394, 480: 394, 510:217, and 297:110 for IM, N-des-methyl imatinib, pyridine–N-oxide imatinib and palonosetron (IS), respectively.

### Calibration Curve

Stock solutions of IM, N-des-methyl imatinib, pyridine-N-oxide imatinib, and palonosetron as internal standard (IS) were prepared by dissolving 1 mg of each drug in 1 ml methanol/water (50:50). Serial dilutions were prepared at concentrations that ranged from (4.8–5,000) ng/ml for IM, (2.7–700) ng/ml for N-des-methyl imatinib, and (5.4–700) ng/ml for pyridine-N-oxide imatinib in drug-free plasma and spiked with the known concentration of the IS as previously described in sample predation calibration plasma standards of imatinib and its metabolites were constructed and used for sample calculation.

### Pharmacogenetic Analysis

#### Preparation of DNA

For determination of polymorphism, Leukocyte cell pellets were isolated using hemolysis buffer (8.46 g ammonium chloride, 1 g ethylene diamine disodium salt, and 1 g potassium bicarbonate dissolved in 1 L at the pH = 7–7.2). The whole-cell pellet was used for the extraction of genomic DNA using the Gentra Puregene Blood Kit (Qiagen Inc, Minneapolis, MN, USA) following the manufacturer's instructions. The DNA was stored at −80°C until analysis.

Polymerase chain reaction-restriction fragment length polymorphism (PCR-RFLP) method was adopted for the genotyping of ABCG2 SNPs 34 G>A (rs2231137) and 421C >A (rs2231142), ABCB1 SNPs 2677 G>A/T (rs2032582), 2677 G> T/A (rs2032582), 1236 C>T (rs1128503), 3435C>T (rs60023214), the solute carriers OATP1B3 (SLCO1B3) SNPs 334T>G (rs 4149117) and CYP 450 3A5 (rs776746). Thermal cycling was started using a thermocycler (Biometra, Germany) according to parameters described in Supplement 1.

Ten μl of each sample was analyzed, along with a suitable DNA size marker, by electrophoresis on 2.5% agarose/ethidium bromide gel. The gel was photographed by (Uvitec, Cambridge, UK) (26, 27).

### Assessment of Drug Adherence

MMAS is a validated, self-reported questionnaire that is a commonly used tool to assess a person's medication adherence behavior. The MMAS is a 4–8 question survey that is designed to measure a specific medication-taking behavior. The use of the modified MMAS is common in the biomedical and professional literature and is customized to address circumstances surrounding adherence behavior related to a particular disease state. Scoring is based on yes/no answers that are then scored by an administrator. Score and intent to adhere to a prescribed medication regimen share a direct relationship (28).

### Statistical Method

Genotype distribution was tested for Hardy-Weinberg equilibrium (HWE) using Fisher's exact test. Quantitative variables were described by mean ± standard deviation and median with range are used when distribution did not follow normality. Qualitative data was described by number and percentages and Chi-square or Fisher exact tested proportion independence. For comparing mean values of 2 independent groups, a *t*-test was used, and for more than 2 groups, we used a one-way analysis of variance (ANOVA) test followed by Bonferroni test for “pairwise group comparisons.” Relation analysis was used to show the strength and significance of the association between quantitative variables. Logistic regression analysis showed which of the variables that relate to unfavorable response has an independent effect on the response to chemotherapy. *P*-value is always 2 tailed and significant at 0.05 level. Graphs were performed using Prism software program (GraphPad prism software, version 5, CA, USA).

## Results

### The Demographic Characteristics and Treatment Response of CML Patients

Table 1 shows the demographic characteristics of 102 CML patients, and the study includes 47 males and 55 females with a mean age of 40.6 years with performance status (0 and 1) with normal liver and kidney laboratory functions. Only 7.8% of the patient has Eutos score ≥87 which indicating high risk. When correlating the demographic characteristics with the response, the only significant relation was between blood platelets and the response (*P* = 0.047). During the two years follow-up, 37 (36.6%) patients showed an unfavorable response (includes patients who do not achieve a molecular response and the patients who develop a progressive disease as an accelerated phase or blast crises). While the favorable responses were achieved in 64 (63.6%) patients and include patients who achieved complete molecular response (CMR), major molecular response (MMR) or complete cytogenetic response (CCyR) (Table 2).

**Table 1:** Patient characteristics and response to therapy.

**Characteristics**		**Total (*N* = 102)**	**Favorable response (*N* = 64)**	**Unfavorable response (*N* = 37)**	***P*-value**
**Gender**					0.636
Male	N (%)	47.0 (46.0%)	28 (43.8%)	18 (48.6%)	
Female	N (%)	55.0 (53.9%)	36 (56.3%)	19 (51.4%)	
Age (year)	Mean (SD)	40.6 (11.0)	40.9 (10.0)	40.0 (12.6)	0.629
**PS**					0.687
0	N (%)	99.0 (97.0%)	63 (98.4%)	35 (94.6%)	
1	N (%)	3.0 (2.9%)	1 (1.6%)	2 (5.4%)	
**Smoking**					0.093
Current smoker	N (%)	13 (12.7%)	5 (7.8%)	7 (18.9%)	
No smoking	N (%)	89 (87.2%)	59 (92.2%)	30 (81.1%)	
**Other diseases other than CML**	N (%)	35 (34.3%)	23 (35.9%)	12 (32.4%)	0.723
Hypertension	N (%)	8 (7.8%)			
Diabetes	N (%)	8 (7.8%)			
Others	N (%)	21 (20.5%)			
Administration of concomitant Drug	N (%)	26 (25.5%)	16 (25.0%)	10 (27.0%)	0.823
					0.938
Family history of cancer	N (%)	16 (15.7%)	10 (15.6%)	6 (16.2%)	
**Eutos score**					0.1
High risk	N (%)	8 (7.8%)	3 (4.7%)	5 (13.5%)	
Low risk	N (%)	94 (92.2%)	61 (95.3%)	32 (86.5%)	
**Laboratory data at sampling time**					
Hg (g/dl)	Mean (SD)	11.7 (1.9)	11.7 (1.7)	11.5 (2.1)	0.857
WBC (/mm^3^)	Mean (SD)	5.7 (2.2–185.0)	5.7 (2.5–15.5)	5.6 (2.2–185.0)	0.413
platlet (/mm^3^)	Median (Range)	186.5 (14.0–934.0)	197.5 (100–934)	152.5 (14.0–554)	0.047*
AST (IU)	Median (Range)	22 (11.7–115.4)	22.2 (12.0–68.0)	21 (11.7–115.4)	0.302
ALT (IU)	Median (Range)	18.2 (7.4–117.0)	19.0 (8.0–72.3)	17.6 (7.4–117.0)	0.766
Creatinine(mg/dl)	Median(Range)	0.9 (0.3–1.4)	0.9 (0.49–1.4)	0.8 (0.3–1.3)	0.117
Urea (mg/dl)	Median (Range)	24.0 (12.0–46.0)	23.6 (12.0–46.0)	24.0 (12.0–41.0)	0.884

Hg, Hemoglobin; WBC, White Blood cell Count; PS, Performance Status; AST, Aspartate, aminotransferase; ALT, Alanine aminotransferase; SD, Standard Deviation; Eutos score: 7*basophils%+4*spleen size (distance from costal margin cm), high risk ≥ 87, low risk ≤ 87.

**Table 2:** Drug Doses and Response.

**Characteristics**		***N* = 102**
**Imatinib treatment dose**		
400 mg	No of patients (%)	94 (92.1%)
300 mg	No of patients (%)	7 (6.8%)
200 mg	No of patients (%)	1 (0.98%)
**BCR/ABL at sampling time**		
Unfavorable Response	Mean (SD)	0.73 (1.37)
PD	Mean (SD)	1.45 (0.05)
NR	Mean (SD)	0.70 (1.38)
Favorable response	Mean (SD)	0.007 (0.01)
CMR	Mean (SD)	0 (0)
MMR	Mean (SD)	0.002 (0.002)
CCyR	Mean (SD)	0.04 (0.03)
**Response after 1 year of follow-up**		
Unfavorable Response	No of patients (%)	37 (36.6%)
PD	No of patients (%)	3 (8%)
NR	No of patients (%)	34 (92%)
Favorable response	No of patients (%)	64 (63.4%)
CMR	No of patients (%)	21 (33%)
MMR	No of patients (%)	34 (53%)
CCyR	No of patients (%)	9 (14%)
**Response after 2 years of follow-up**		
Unfavorable response	No of patients (%)	37 (36.6%)
PD	No of patients (%)	3 (8%)
NR	No of patients (%)	34 (92%)
Favorable response	No of patients (%)	64 (63.4%)
CMR	No of patients (%)	21 (33%)
MMR	No of patients (%)	32 (50%)
CCyR	No of patients (%)	11 (17%)

*SD, Standard Deviation; FR, Favorable Response; UF, Unfavorable Response; PD, Progressive Disease; NR, No Response; CMR, Complete Molecular Response; MMR, Major Molecular Response; CCyR, Complete Cytogenetic Response*.

### Drug Level and Pharmacokinetic Data

The IM pharmacokinetic is considered as a one-compartment model. This model considers all body tissues and fluids as a part of this compartment. It assumes that the dose of drug distributes instantaneously to all body areas and a rapid drug equilibrium between the blood and tissue distribution and elimination occurs. In this study we used the formulae to calculate pharmacokinetic parameters using two points, the trough and the peak (29). Table 3 shows the levels of pharmacokinetic parameters of IM. The plasma IM peak level was 2,423 ± 902 ng/ml (mean ± SD), while the trough plasma level was 1,199 ± 506 ng/ml and peak/trough ratio (P/T) ratio were 2.4 ± 1.5. The peak concentration of the active N-des-methyl imatinib plasma level was 311.1 ± 129.2 ng/ml; the trough concentration was 208.8 ± 98.2 ng/ml. The second metabolite, pyridine -N-oxide imatinib, had a peak concentration of 37.0 ± 22.0 ng/ml and the trough concentration was 16.7 ± 12.6 ng/ml. Following the one-compartment model, the pharmacokinetic parameters of IM were the median (range), 0.034 (0.0005–0.11), the mean concentrations of steady (C_SS_), volume of distribution (V_d_) and clearance (Cl) of IM were 1,769 ± 689 ng/ml, 186 ± 81.7 L and 158 ± 147 L/h., respectively, Table 3.

**Table 3:** Relation of Response with Pharmacokinetic Parameters.

	**Unit**	**Total**	**Favorable response (*N* = 64)**	**Unfavorable response (*N* = 37)**	***P*-value**
		**(Mean** **±** **SD)**	**Mean** **±** **SD**	**Mean** **±** **SD**	
**IM level**					
Peak	ng/ml	2,423 ± 902	2,378 ± 893	2,433 ± 927	0.45
Trough	ng/ml	1,199 ± 506	1,281 ± 578	935 ± 559	0.006^*^
P/T ratio		2.4 ± 1.5	2.06 ± 0.8	2.8 ± 2.1	0.001^*^
**N-des-methyl imatinib**					
Peak	ng/ml	311.1 ± 129.2	306 ± 131	320 ± 128	0.61
Trough	ng/ml	208.8 ± 98.2	217 ± 94	192 ± 104	0.22
P/T ratio		1.71 ± 1.33	1.51 ± 0.52	2.13 ± 2.09	0.095
**Pyridine-N-oxide imatinib**					
Peak	ng/ml	37.1 ± 22.0	38.2 ± 24.3	35.5 ± 17.2	0.52
Trough	ng/ml	16.8 ± 12.6	18.8 ± 14.2	13.2 ± 8.1	0.01^*^
P/T ratio		2.78 ± 2.13	2.06 ± 0.79	3.12 ± 2.09	0.008^*^
**Pharmacokinetic parameters**					
K_e_		0.034 (0.0005–0.11)	0.029 ± 0.018	0.040 ± 0.021	<0.001^*^
C_SS_	ng/ml	1,769 ± 689	1,840 ± 679	1,684 ± 706	0.22
V_d_	L	186 ±81.7	192 ± 89	166 ± 64	0.41
Cl	L/hr	158 ± 147	128 ± 137	175 ± 158	0.004^*^
Alph-1-acid gp	μg/ml	1.62 ± 0.52	1.62 ± 0.55	1.58 ± 0.44	0.68

* *P value is significant ≤ 0.05*.

*IM, Imatinib; K_e_, elimination constant; C_SS_, IM Steady State plasma Concentration; V_d_, Volume of distribution; Cl, IM Clearance; P/T, Peak/Trough ratio*.

### Relation of Pharmacokinetic Parameters and Clinical Response

**Figure 1:**
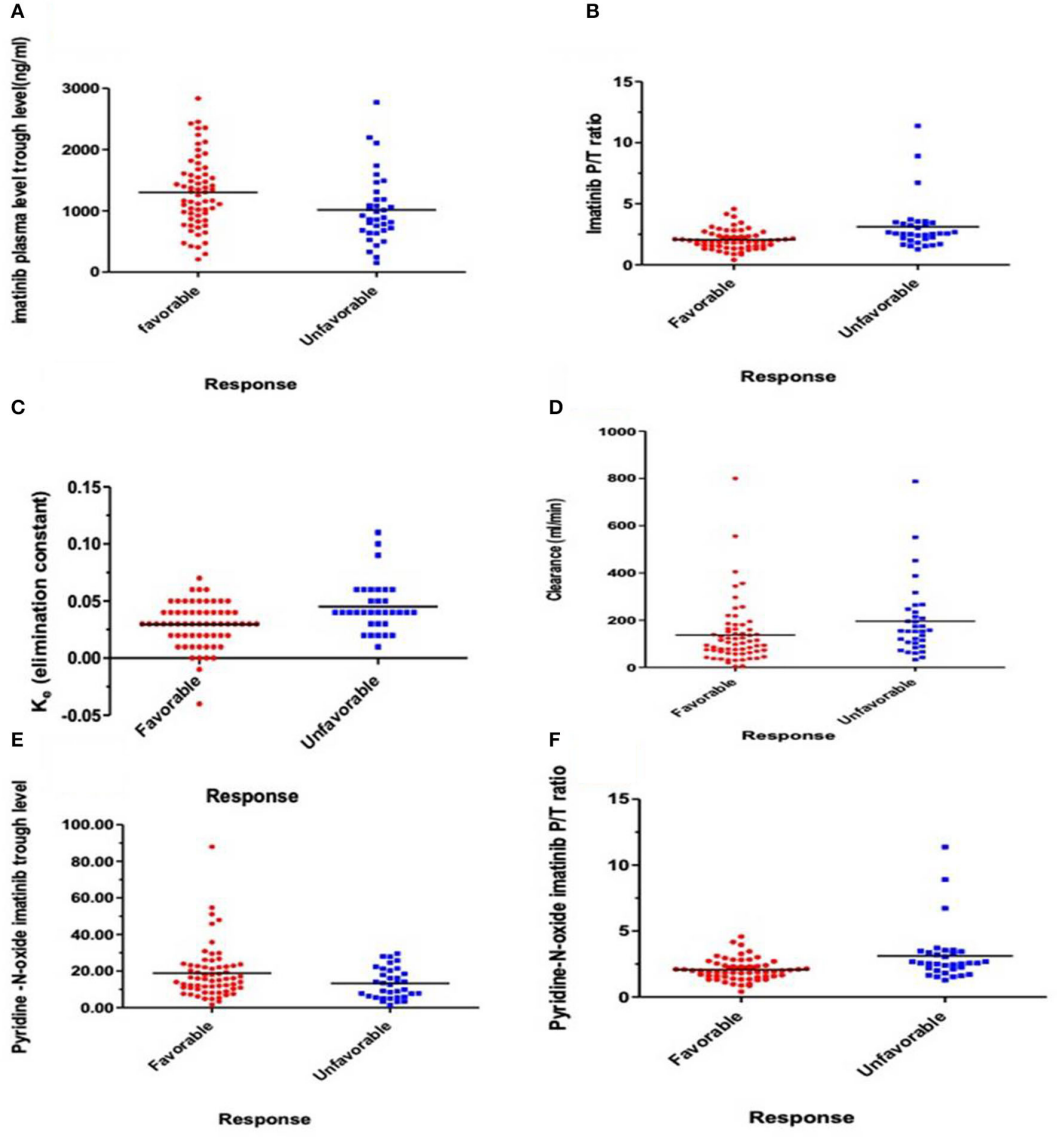
Relations express the relation between response and pharmacokinetic parameters. **(A)** Plasma IM trough concentration (ng/ml) (*P* = 0.006), **(B)** IM peak/trough ratio (P/T) (*P* = 0.001), **(C)** Elimination rate constant (K_e_) (*P* = 0.001), **(D)** IM clearance (ml/min) (*P* = 0.004), **(E)** Plasma trough of Pyridine-N-oxide imatinib (*P* = 0.01), and **(F)** Peak/Trough ratio (P/T) Pyridine-N-oxide imatinib (*P* = 0.008).

Logistic regression analysis was done where the favorable or unfavorable response was as the dependent variable and age, sex, IM dose, and trough level P/T ratio of IM and pyridine -N- oxide, K_e_, and clearance as prognostic variables. The relation between different pharmacokinetic parameters and the clinical response to IM therapy is presented in Table 3 and Figure 1. There was an insignificant relation between the demographic characteristics and IM dose with the response. The IM trough level among patients who achieved favorable response (*N* = 64) was significantly higher than those for patients who did not achieve favorable response (*N* = 37). The mean values of the trough level were 1,281 ± 578 ng/ml for responders vs. 935 ± 559 ng/ml for non-responders, respectively (*P* = 0.006). The P/T ratio of patients who achieved favorable response was significantly lower (2.06 ± 0.8) than those for patients who had an unfavorable response (2.8 ± 2.1) (*P* = 0.001) (Table 3 and Figure 1). Similarly, the trough concentration of pyridine-N-oxide was significantly higher in patients who had favorable responses (*P* = 0.01). The elimination rate constant and the clearance are significantly higher in patients who achieve unfavorable responses (*P* = 0.001 and 0.004), respectively. After logistic regression analysis, the only significant independent factor affecting the response was the P/T ratio of IM. As the peak/trough ratio increased by one, the risk of bad response increased by more than double as compared to favorable response with 95% CI (1.28–3.92, *P* = 0.005).

### Relation Between Genetic Polymorphism and Clinical Response

**Figure 2:**
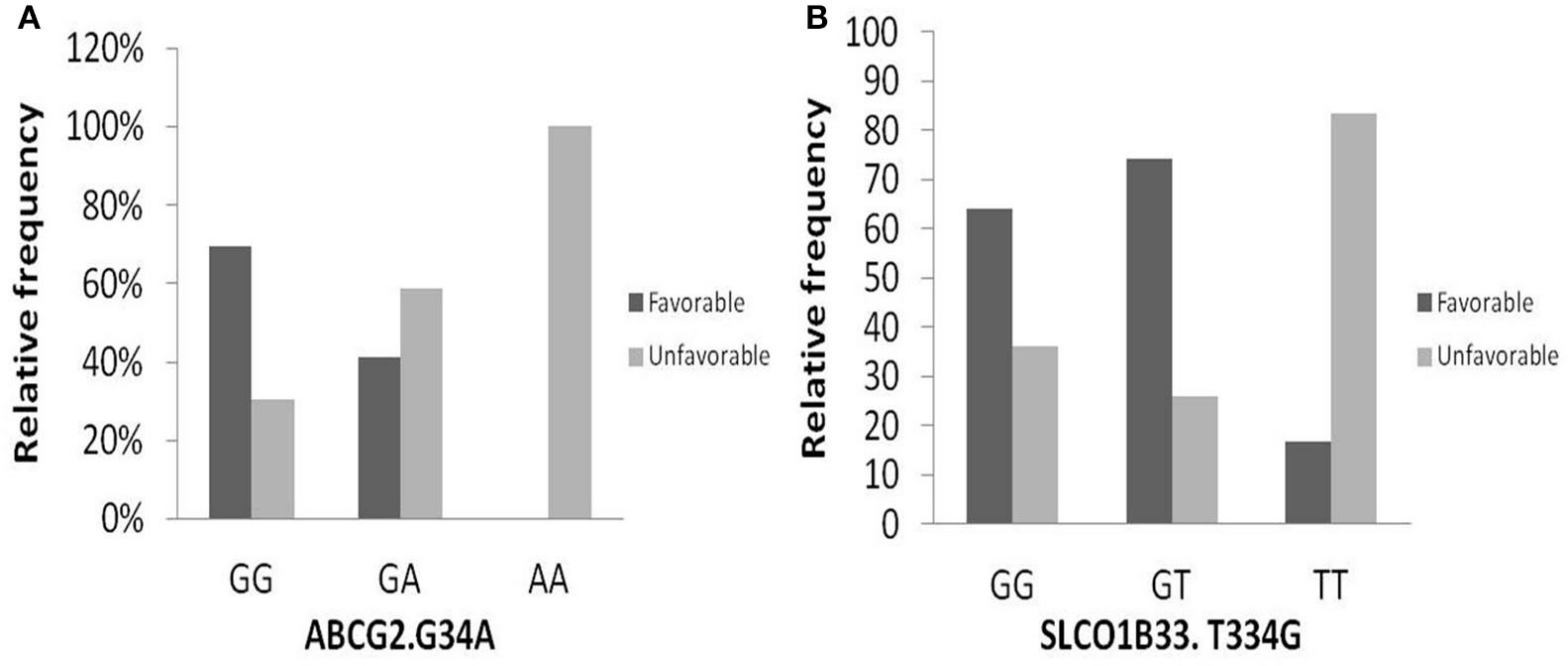
Relation of relative frequency of the gene variant ABCG2. 34 G>A and SLCO1B3. 334 T>G with response. **(A)** ABCG2. 34 G>A gene, **(B)** SLCO1B3. 334 T>G gene.

**Figure 3:**
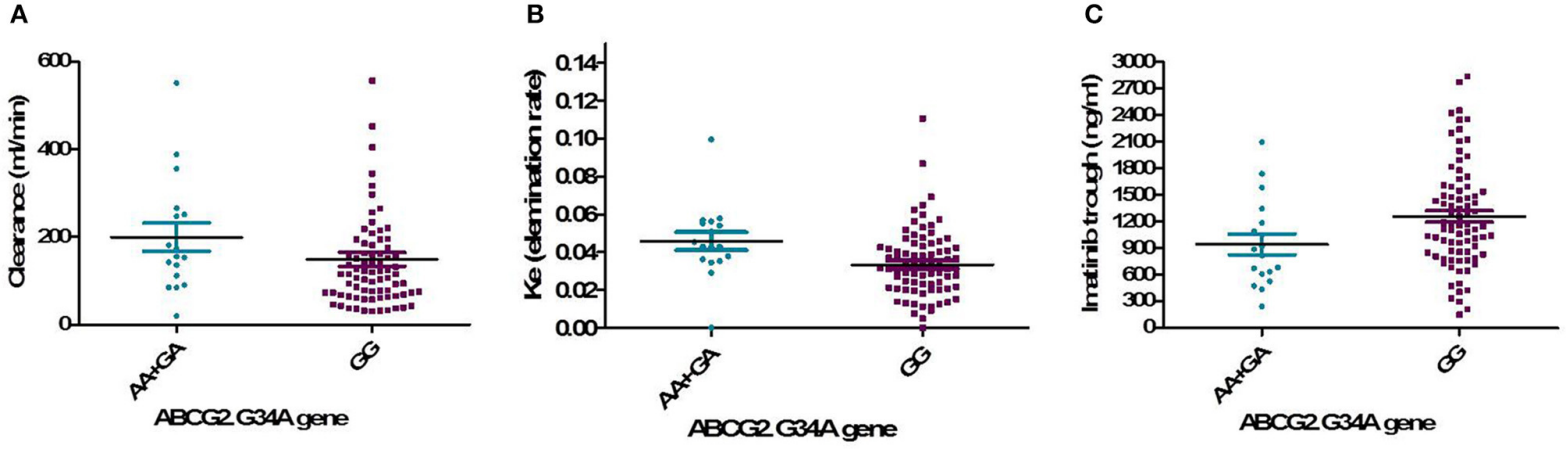
Relation of the genotype variant ABCG2. 34 G>A gene with pharmacokinetic parameters. **(A)** IM clearance (*P* = 0.02), **(B)** K_e_, elimination rate constant (*P* = 0.005), **(C)** IM trough plasma concentration (*P* = 0.01).

One hundred and two DNA samples were analyzed using PCR-RFLP displayed in Supplement 2 and Logistic regression analysis was done where favorable or unfavorable response as was the dependent variable and gene polymorphism were the prognostic variables in the model, Table 4; Figures 2, 3. The frequency distribution of the genes and their relation with response is illustrated in Table 4. The frequency distribution of the gene variant of the efflux transporter ABCG2.34 G>A among our patients was 81.4% with homozygous wild type GG allele, 16.7% with heterozygous GA allele, and 2% with the variant type AA, Table 4. Regarding the relation of the variant ABCG2. 34 G>A gene with response, it was found the patients with the wild allele GG had higher IM trough level (*P* = 0.01) lower Ke (*P* = 0.005) and clearance (Cl) (*P* = 0.02) Figure 3 and linked to favorable response compared to (GA and AA alleles) (Table 4).

**Table 4:** Relation between Genetic Polymorphism and Clinical Response.

		**The frequency**	**Response**		***P* value**
			**Favorable N (row %)**	**Unfavorable N (row %)**	
ABCG2.34 G>A	GG	83 (81.4%)	57 (69.5%)	25 (30.5%)	0.01^*^
	GA	17 (16.7%)	7 (41.2%)	10 (58.8%)	
	AA	2 (2%)		2 (100.0%)	
ABCG2. 421C>A	CC	91 (89.2%)	55 (60.4%)	36 (39.6%)	0.20
	CA	10 (9.8%)	8 (88.9%)	1 (11.1%)	
	AA	1 (1%)	1 (100.0%)		
ABCB1.2677 G>A/T	GG	101(99%)	64 (64.0%)	36 (36.0%)	0.37
	GA	1 (1%)		1 (100.0%)	
	AA	0 (0%)			
	TT	0 (0%)			
ABCB1.1236 C>T	CC	17 (16.7%)	10 (58.8%)	7 (41.2%)	0.47
	CT	49 (48%)	34 (69.4%)	15 (30.6%)	
	TT	36 (35.3%)	20 (57.1%)	15 (42.9%)	
ABCB1.3435C>T	CC	38 (37.3%)	21 (56.8%)	16 (43.2%)	0.51
	CT	48 (47.1%)	33 (68.8%)	15 (31.3%)	
	TT	16 (15.7%)	10 (62.5%)	6 (37.5%)	
SLCO1B3.334 T>G	TT	7 (7.1%)	1 (16.7%)	5 (83.3%)	0.03^*^
	TG	31 (31.3%)	23 (74.2%)	8 (25.8%)	
	GG	61 (61.6%)	39 (63.9%)	22 (36.1%)	
CYP3A5	^*^1	6 (5.9%)	3 (50.0%)	3 (50.0%)	0.83
	^*^1/3	28 (27.5%)	18 (64.3%)	10 (35.7%)	
	^*^3	68 (66.7%)	43 (64.2%)	24 (35.8%)	

*G, guanine; C, cytosine; A, adenine; T, thiamine; ABCB1, multidrug resistant gene; ABCG2, breast cancer resistant gene; SLCO1B3, uptake gene; CYP3A5, cytochrome metabolizing gene*.

* *p value is significant ≤ 0.05*.

**Figure 4:**
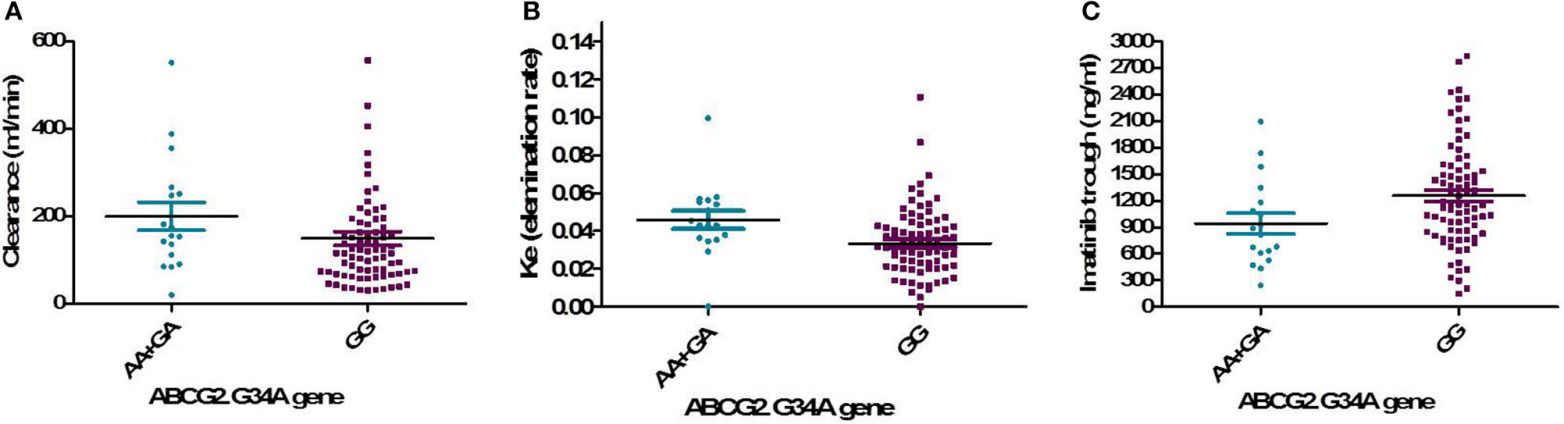
Relation of the genotype variant ABCG2. 421C >A with pharmacokinetic parameters. **(A)** Plasma IM peak concentration (ng/ml) (*P* = 0.03), **(B)** IM steady state concentration (ng/ml) (*P* = 0.05), **(C)** Peak of N-des-methyl IM (ng/ml) (*P* = 0.02).

On the other hand, the frequencies of ABCG2. 421C >A were 89.2, 9.8, and 1% for the CC wild type, the CA heterozygous, and the AA variant, respectively. Concerning the genotype 421C >A, the homozygous CC had significantly higher peak, and C_SS_ of IM as well as higher peak level of desmethyl imatinib compared to the allele AA and CA (*P* = 0.03, *P* = 0.05, *P* = 0.02, respectively), Figure 4.

**Figure 5:**
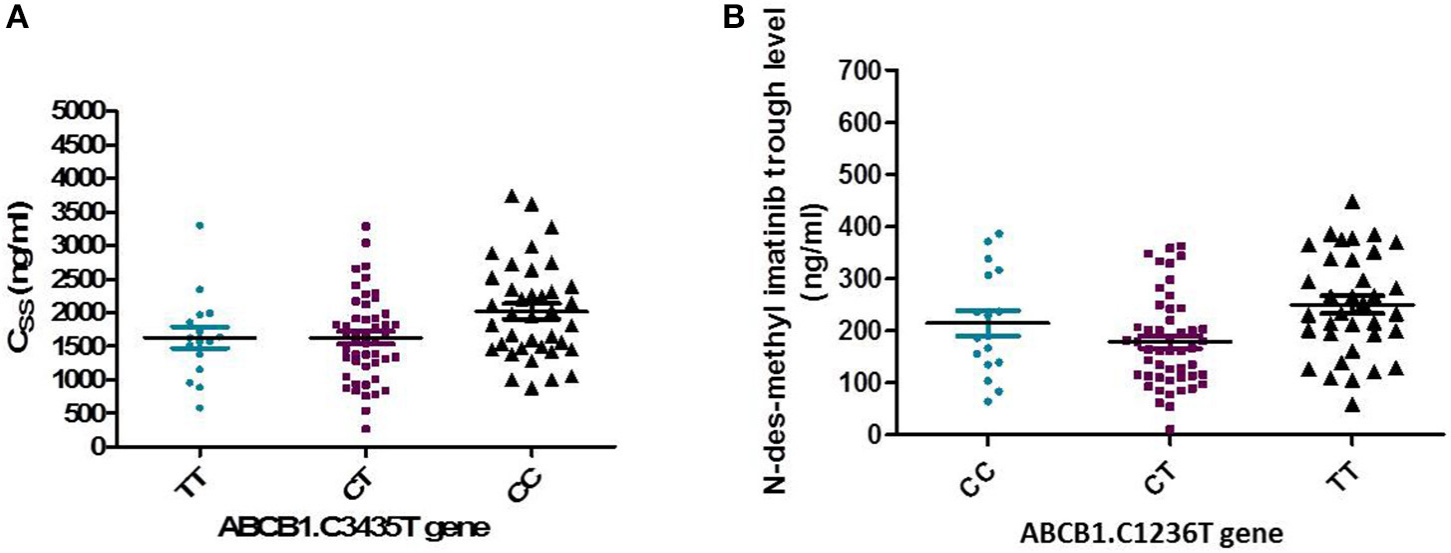
Relation of ABCB1. 3435C>T and 1236 C>T gene with pharmacokinetics of Imatinib. **(A)** Association of ABCB1. 3435C>T with the steady state concentration (ng/ml) (*P* = 0.038). **(B)** Association of ABCB1. 1236 C>T gene with trough concentration of N-des-methyl imatinib (ng/ml) (*P* = 0.004).

The frequency distribution of the efflux transporter gene variant of ABCB1. 3435C>T was 37.3% for the homozygous wild type CC, 47.1% for the heterozygous (CT), and 15.7% for the TT variant type. The wild allele CC of ABCB1.3435C>T gene had significantly higher steady-state concentration compared to the two allele TT and CT (*P* = 0.038), Figure 5A.

In addition, the distribution of ABCB1. 1,236 C>T was 35.3% for TT allele, 48% CT, and 16.7% for CC allele. The relation of 1,236 C>T gene polymorphism with pharmacokinetic parameters, a significant lower N-des-methyl imatinib trough plasma level in heterozygous CT genotype compared to homozygous CC and TT (*P* = 0.004), Figure 5B.

On the other hand, the frequency distribution of homozygous wild type GG of ABCB1.2677 G>A/T was 99%, and the heterozygous frequency GT was 1%, and no patients with the homozygous variant type (AA or TT) were detected, Table 4.

The intake transporter SLCO1B3.334 T>G, known as organic anion-transporting polypeptide 1B3 (OATP1B3), had the following distribution: 7.1% TT, 31.3% TG, and 61.6% GG. The alleles GG and TG of the gene variant SLCO1B3. 334 T>G were significantly correlated to favorable response while wild allele TT was linked to unfavorable response (*P* = 0.03), Figure 2.

In the genotypes of metabolizing enzyme CYP3A5, the frequency distribution was as follows: the homozygous wild variant (^*^1) 5.9%, heterozygous (^*^1/3) 27.5%, and the homozygous variant (^*^3) 66.7%.

Our results did not detect association of the three variants of ATP-binding cassette, subfamily B1 and ABCG2 421C >A, and metabolizing enzyme CYP3A5 with IM response.

### Relation of Adherence and Response

Parametric relation analysis of drug response according to adherence score was illustrated in Supplement 3. The median (range) of MMAS knowledge in the patients who achieved favorable response and the patients who suffering unfavorable response was the same 3 (1–3), the MMAS motivation was 3 (1–3), and 2 (1–3), respectively, and the total adherence score was the same 5 (2–6) with no statistical significance.

### The Most Recorded Side Effects of IM

The recorded side effects of the administered dose of IM was illustrated in Supplement 4. Our result showed 29 (28.4%) patients did not suffer from any side effect; however, the most common side effects were gastrointestinal disorder 24 (23.4%), followed by musculoskeletal disorders 21 (20.5%) and nervous system disorders 21 (20.5%). The gastrointestinal disorders include heartburn 3.0 (2.9%), tooth pain 4.0 (3.9%), visceral pain 15.0 (14.7%), and vomiting 2.0 (1.9%). In addition to fatigue, inflammation disorders, weight were recorded in 7 (6.8%), 4 (3.9%), and 4 (3.9%), respectively.

## Discussion

Imatinib is the current treatment standard of care for CML disease; it induces durable responses and prolongs event and progression-free survival (30). The individual variability in IM pharmacokinetics often leads to an unsatisfactory clinical outcome in patients with CML (31). The results of the present study added a great value to the importance of implementing routine therapeutic drug monitoring (TDM) for better management of CML patients treated with IM for dose adjustment. Data of the present study showed a significant higher trough IM (1281 ng/ml) (*P* = 0.006), lower P/T ratio (*P* = 0.001), lower clearance (Cl) (*P* = 0.004), and elimination rate constant, k_e_ (*P* = 0.001), among patients who achieved favorable responses (*N* = 64) than those for patients who suffered unfavorable responses (935 ng/ml) (*N* = 37). It was reported that patients with trough concentration over 1,002 ng/ml of IM were good responders and were significantly associated with achieving MMR (32–34). Contrary to the previous studies, IM trough concentration was not significantly associated with the molecular response in Chinese CML patients (35) that was referred to ethnic metabolic distinction of Chinese compared to Caucasian CML patients group (36).

The present study is the first to report an association of Peak/Trough ratio of IM with the response. The P/T ratio was the only significant independent factor affecting the response, as the P/T ratio increased by one, the risk of unfavorable response increased by more than double as compared to favorable response with 95% CI (1.28–3.92, *P* = 0.005). Moreover, like our results of IM, the trough concentration of Pyridine-N-oxide imatinib was significantly higher (*P* = 0.01) and its P/T ratio was significantly lower (*P* = 0.008) in patients achieved favorable response than those without. Up until now, we did not find any previous data about the biological activity or the relation of the level of this metabolite with the response to IM. However, limited information about the biological activity of pyridine-N-oxide metabolite as it has some activity, we suggest that the Pyridine-N-oxide imatinib may be a final product due to the action of other types of cytochromes on IM or it may pass certain pathways that lead to its activation. In addition, great variability in Cl, the volume of distribution, and elimination half-life (T_1/2_) of IM were reported in previous studies as well as in our study. The variability in Cl of IM was ~73% and the volume of distribution was 63% (37). These differences in pharmacokinetics parameters may be related to demographic covariates as hepatic and renal dysfunction, body weight, age, sex, and disease state (38).

The interindividual Pharmacogenetic variability can affect IM disposition and its metabolism leading to differences in drug responses (7). The ABCG2 gene encodes a membrane-bound protein and acts as a xenobiotic transporter which may have a role in drug resistance to chemotherapeutic agents, including IM (14). In this study, the distribution frequency of ABCG2 the genotype 34 G>A was GG (81.4%), GA (16.7%), and AA (2%), and the GG allele was associated with favorable response (*P* = 0.01), lower Cl, K_e_, and higher trough IM than both (AA+GA) alleles. Our result was supported by the study of Delord et al. (15) who reported the GG genotype had favorable response compared to genotypes AA and GA. On the other hand, a Canadian study found that the GG genotype 34 G>A was significantly correlated with unfavorable response to IM especially for CCyR (16). These diversities of findings may be due to ethnic variability.

The current data showed that the wild allele (CC) of the genotype ABCG2 421C >A had significantly higher plasma peak of IM (*P* = 0.03), higher C_ss_ (*P* = 0.049), and higher N-des-methyl imatinib (*P* = 0.02). However, a previous study found that the CC genotype was higher significantly in IM resistant group (*P* = 0.004), and the CA genotype was higher with favorable response (*P* = 0.0001) (26). Moreover, Takahashi et al. (13) found that AA of the genotype had significantly higher IM trough concentration (*P* = 0.015), while Jiang et al. (34) disclosed a relation of patient with AA genotype and higher rate of MMR.

The ABCB1 gene, one of the ABC superfamily, encodes for a transmembrane glycoprotein (P-glycoprotein) capable of pumping IM out of the tumor cell (12). Its overexpression may offer resistance to IM in the cell lines model and at the clinical level (17). In the current work, the distribution frequency of the gene ABCB1.3435C>T among our patients was 37.3% (CC), 47.1% (CT), and 15.7% (TT), and the variant TT and CT had significantly lower C_ss_ IM compared to CC genotype (*P* = 0.038). No significant relation was detected between the ABCB1 3435C>T and response. Similarly, previous studies of Kim et al. (16), Polillo et al. (18), and Jiang et al. (34) did not find a relation of the genotype 3435C>T and response. Contrary to the previous studies and our results, Angelini et al. (19) denoted a significant association of (CC) genotype with CMR in Caucasians patients while the TT genotype was higher in IM resistant CML patients (26). Moreover, patients with the (CC) genotype were found to have significantly higher IM clearance (*P* = 0.035) (20) and had a poor cytogenetic response (21).

Our data revealed the variant ABCB1.1236 C>T had a distribution frequency of 16.7% (CC), 48%CT, 35.3%TT, a significantly higher Css (ng/ml) in CC carriers than those carrying the homozygous TT genotype and the heterozygous CT genotype (*P* = 0.038). The heterozygous CT variant of ABCB1. 1236 C>T, had significantly lower N-des-methyl imatinib trough than CC and TT (*P* = 0.004). On the other hand, a previous study reported an association between 1236 C>T (TT/CT) and higher MMR rates (22). The variations in frequencies are ethnically related (39).

Concerning the frequency distribution of the ABCB1 2677 G>A/T gene, it showed 99% (GG), and 1% (GA). Our result is in agreement with a previous study that revealed a significantly lower frequency of TT genotype in Egyptian CML patients. On the contrary to our results, they reported optimal and suboptimal responses in patients with TT genotype; however, GG genotype had no association with drug response (40). It was suggested that these contradictions may be due to the presence of different amino acids at position 893. The SNP 2677 G>A/T (TT) in exon 21 (893 codon) results in substitution of alanine lipophilic residue to serine/threonine hydrophilic residue resulting higher resistance to various drugs such as Adriamycin and vinblastine (41).

The organic anion transporting polypeptides OATP1B3 was suggested to mediate the uptake of IM into cells. Therefore, it is suggested that *SLCO1B3*.334 T>G genotype may have a clinical importance in IM treatment outcome prediction. Our data showed a significant association of the genotype TT of SLCO1B3 334 T>G with higher risk of unfavorable response (*P* = 0.03), while the patients with (GT/GG) genotypes showed a higher probability of achieving a favorable response (*P* = 0.03). Our finding agrees with the study of Nair et al. (42). Contrary to the previous result, the (TT) genotype was more frequent in the responder group (*P* = 0.042) and carriers of (TG/GG) genotypes were more frequent in the non-responder group (43). Moreover, no association was found between SLCO1B3.334 T>G polymorphism and response to IM therapy (*P* = 0.938) (27).

The genotype frequency fraction of the CYP3A5 gene is different in different ethnic populations. In Japanese it was 23.25% (1^*^), 76.75% (3^*^), in Caucasian it was 14.81% (1^*^), 85.19% (3^*^). In our study, the genotype distribution of CYP3A5 was 5.9% for (1^*^) allele, 27.5% for (1/3^*^) allele, 66.7% for (3^*^) allele. Our data are supported by previous Egyptian study found that the fraction of (^*^3) allele was (50%) and the (^*^1) allele was the least frequent (12.5%). Although the authors found that the CYP3A5^*^3 gene polymorphism was linked to unfavorable outcome (*P* < 0.001) (27), contrary to our result that did not detect an association between CYP3A5 gene as and pharmacokinetic parameters or response. Similarly, to our data, Takahashi et al. (13) and Ankathil et al. (44) reported insignificant relations between CYP3A5 gene polymorphism and IM trough concentration (*P* = 0.645). Despite the important role of CYP activity in the regulation of IM metabolism, it may be due to its activity and not the rate-limiting step in IM metabolism and excretion (45).

In conclusion, the current study is the first to disclose the novel relations of the high trough, a low P/T ratio of IM and its metabolite, pyridine -N-oxide imatinib to good IM response, as well as to reveal that the only independent prognostic marker IM response is P/T ratio of IM. Moreover, the association of the genotypes ABCG2.34 G>A and SLCO13.334 T>G with IM favorable response in Egyptian CML patients.

Future studies with larger patients' numbers are necessary to validate these findings, including the assessment of patients' compliance with more than one method, confirmation of the different types of genotypes of polymorphism by gene expression, as well as recording the drug toxicity and its relation with IM kinetics and polymorphism.

## Data Availability Statement

All datasets generated for this study are included in the article/Supplementary Material.

## Ethics Statement

The studies involving human participants were reviewed and approved by the Institution Review Board (IRB) of the National Cancer Institute of Cairo University, Egypt with acceptance number IRB00004025. The patients/participants provided their written informed consent to participate in this study in accordance with the Declaration of Helsinki.

## Author Contributions

MO performed sample collection, the experimental work, and drafted the paper. RA help in patient follow-up and revised the manuscript. HM helped in experimental work and revised the manuscript. NA performed data statistical analysis. SS designed the experimental work and revised the manuscript. All authors contributed to revising the manuscript and approved it.

## Conflict of Interest

The authors declare that the research was conducted in the absence of any commercial or financial relationships that could be construed as a potential conflict of interest.
