# Supplementary material for: Association of the Trough, Peak/Trough Ratio of Imatinib, Pyridine–N-Oxide Imatinib and ABCG2 SNPs 34 G>A and SLCO1B3 334 T>G With Imatinib Response in Egyptian Chronic Myeloid Leukemia Patients
Source: Front Oncol. 2020 Aug 19;10:1348. doi: 10.3389/fonc.2020.01348 (PMC7466443; doi:10.3389/fonc.2020.01348)
Supplement: Supplementary file 1 [file Data_Sheet_1.docx]

**Supplement [1]:** Primers Sequences, Restriction Enzymes and Working Conditions of Genes of Interest.

| Gene | polymorphism | Primer sequence | Anneling temperature (X) | PCR product (bp) | Restriction enzymes | Digestion temperature (Y) | Fagments length after digestion (bp) |
| --- | --- | --- | --- | --- | --- | --- | --- |
| β_2_globin | House keeping gene | forward 5-GGCACACTCCAGGCATAGGTAAAGA-3  reverse 5-TGGAAGAGGCTTCTTCCACCTTGGAA-3 | 60°C | 300-bp | --------- | -------- | --------------------- |
| ABCB1 | 3435C>T  (rs60023214) | forward 5-GATCTGYGAACTCTTGTTTTCA-3  reverse 5-GAAGAGAGAGACTTACATTAGGC-3 | 61°C | 244-bp | Mbo I | 37°C | C allele :172-72  Tallele : 244 |
|  | 1236 C>T  (rs1128503) | forward 5- TTCACTTCAGTTACCCATC-3  reverse 5- CATAGAGCCTCTGCATCA-3 | 49°C | 314-bp | Bsu R I | 37°C | C allele 252- 62  T allele 217- 62- 35 |
|  | 2677 G>A/T  (rs2032582) | forward 5- AGAGCATAGTAAGCAGTAGGGAGTA-3  reverse 5- GCAAATCTTGGGACAGGAATA-3 | 61°C | 256-bp | RsaI | 37°C | G or T allele 183 -73  A allele 101- 82- 73 |
|  | 2677 G> T/A  (rs2032582) | forward 5- AGTAAGCAGTAGGGAGTAACA-3  reverse 5- GATAAGAAAGAACTAGAACGT-3 | 53°C | 114-bp | AclI | 37°C | G or A allele :114  T allele:: 94- 20 |
| ABCG2 | 34 G>A  (rs2231137) | forward 5-CAGTAATGTCGAAGTTTTTATCGCA-3  reverse 5- AAATGTTCATAGCCAGTTTCTTGGA-3 | 59°C | 291-bp | Bse 3D I | 60°C | G allele :291  A allele : 261- 30 |
|  | 421C >A  (rs2231142) | forward 5- GTTGTGATGGGCACTCTGATGGT-3  reverse 5- CAAGCCACTTTTCTCATTGTT-3 | 58°C | 259-bp | Bst 4C I | 65°C | C allele : 259  A allele : 229- 30 |
| SLCO1B3 | 334 T>G  (rs 4149117) | forward 5-GAAGGTACAATGTCTTGGGC-3  reverse 5-CTCTCAAAAGGTAACTGCCC-3 | °64 C 64 | 33339-bp  -b | Alu I | 37°C | T allele:253  G allele :213 |
| CYP3A5 | (rs776746) | forward 5-GTTGTACGCCACACACC-3  reverse 5-CTCTTTAAAGAGCTCTTTTGTCTCTCA-3 | ° 54C 54 | 15155-bp | Dde I | 37°C | CYP3A5*1 (Wild allele): 121-34  CYP3A5*3 (mutant allele) :97-34- 24 |

**
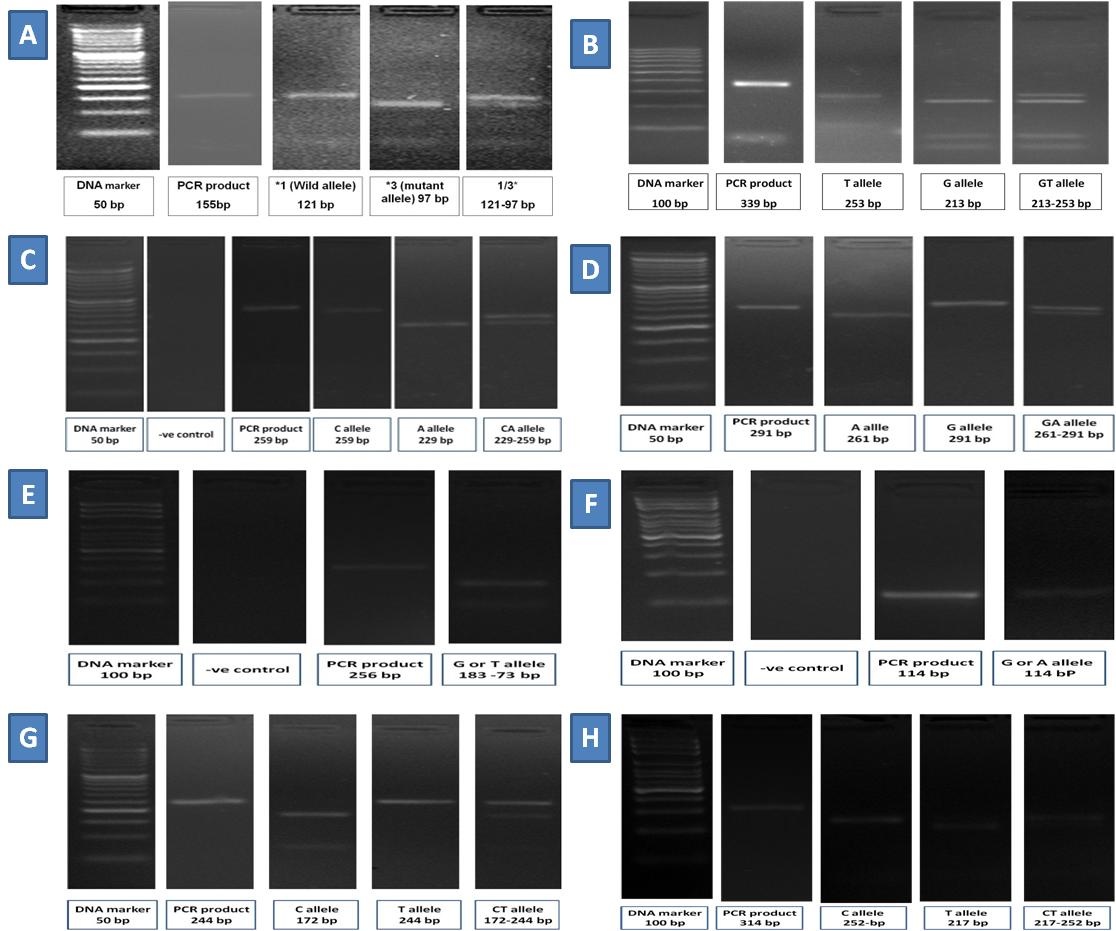
**

**Supplement [2]:** Genotyping of the studied genes using genomic DNA by PCR-RFLP. **A:**CYP 3A5 digested by DdeI enzyme compared to 50 bp marker (*1 : 121,*3: 97), **B:** SLCO1B3. 334 T>G digested by AluI enzyme compared to 100 bp marker (T allele:253,G allele:213), **C:** ABCG2. 421C >A digested by Bst4CI enzyme compared to 50 bp marker (C allele : 259, A allele : 229), **D:** ABCG2.34 G>A digested by Bse3DI enzyme compared to 50 bp marker(G allele :291, A allele : 261), **E/F:**ABCB1. 2677 G>A/T (triallelic gene) digested by RsaI/AclI enzyme compared to 100 bp marker (G or T allele: 183, A allele: 101/ G or A allele: 114,T allele: 94), **G:**ABCB1. 3435C>T digested by MboI enzyme compared to 50 bp marker (C allele :172,T allele : 244), **H:** ABCB1.1236 C>T digested by BsuRI enzyme compared to 100 bp marker (C allele 252,T allele 217).

**Supplement [3]:** Side effects of the administered dose of the drug.

| Side effects | Total of patients (%) | 102 (100%) |
| --- | --- | --- |
| Nervous system disorders |  |  |
| Drowsiness | No of patients (%) | 9.0 (8.8%) |
| Headache | No of patients (%) | 7.0 (6.8%) |
| Numbness | No of patients (%) | 5.0 (4.9%) |
|  |  |  |
| Cardiac disorders | No of patients (%) | 4.0 (3.9%) |
|  |  |  |
| Vascular disorders (Hemorrhage) | No of patients (%) | 3.0 (2.9%) |
|  |  |  |
| Gastrointestinal disorders |  |  |
| Heartburn | No of patients (%) | 3.0 (2.9%) |
| Tooth pain | No of patients (%) | 4.0 (3.9%) |
| Visceral pain | No of patients (%) | 15.0 (14.7%) |
| Vomiting | No of patients (%) | 2.0 (1.9%) |
|  |  |  |
| Skin and subcutaneous tissue disorders |  |  |
| Hair loss | No of patients (%) | 2.0 (1.9%) |
| Rash | No of patients (%) | 4.0 (3.9%) |
| Skin discoloration | No of patients (%) | 6.0 (5.8%) |
| Skin dryness | No of patients (%) | 1.0 (0.98%) |
|  |  |  |
| Musculoskeletal disorders |  |  |
| Bone pain | No of patients (%) | 15.0 (14.7%) |
| Cramps in extremities | No of patients (%) | 6.0 (5.8%) |
|  |  |  |
| Fatigue | No of patients (%) | 7.0 (6.8%) |
|  |  |  |
| Inflammation disorders | No of patients (%) | 4.0 (3.9%) |
|  |  |  |
| Weight change | No of patients (%) | 4.0 (3.9%) |
|  |  |  |
| No side effects | No of patients (%) | 29.0 (28.4%) |
|  |  |  |

- Patients may suffer more than one side effect.

**Supplement [4]: Correlation of Response with Morisky Medication Adherence score.**

| P value | Unfavorable response (N=37) | Favorable response (N = 64) |  |
| --- | --- | --- | --- |
|  |  |  |  |
|  | **Median(range)** | **Median(range)** | MMAS |
| 0.76 | 3 (1-3) | 3 (1-3) | Knowledge |
| 0.30 | 2 (1-3) | 3 (1-3) | Motivation |
| 0.71 | 5 (2-6) | 5 (2-6) | Total adherence score |

**Abbreviations:** MMAS: Morisky Medication Adherence Scores.
